# Effect of platelet rich fibrin on edema and pain following third molar surgery: a split mouth control study

**DOI:** 10.1186/s12903-017-0371-8

**Published:** 2017-04-24

**Authors:** Uğur Gülşen, Mehmet Fatih Şentürk

**Affiliations:** 1 Department of Oral and Maxillofacial Surgery, Faculty of Dentistry, Bulent Ecevit University, Zonguldak, Turkey; 2 Department of Oral and Maxillofacial Surgery, Faculty of Dentistry, Süleyman Demirel University, 32260 Çünür, Isparta Turkey

**Keywords:** Impacted third molar surgery, Platelet rich fibrin(PRF), Edema, Pain

## Abstract

**Background:**

To evaluate the efficacy of platelet-rich fibrine (PRF) on postoperative edema and pain after impacted mandibular third molar surgery.

**Methods:**

The prospective study was comprised 30 patients who presented for the removal of bilateral impacted mandibular third molar teeth. After extraction, the sockets were filled with PRF or without PRF in the study and control groups, respectively. Postoperative edema was measured with a flexible tape measure by calculating the distance between several facial landmarks on postoperative days two and seven. Postoperative pain was evaluated with a line-type visual analogue scale (VAS) and a verbal scale (VRS). SPSS version 20.0 was used for data analysis.

**Results:**

Both groups recorded significant improvement compared to the baseline levels in almost all of the outcome variables. There was no statistically significant difference between the study and control groups (*p* > 0.05).

**Conclusions:**

Using or not using PRF to reduce postoperative pain and edema in third molar surgery was equally successful.

**Trial registration:**

This study was retrospectively registered at the ISRCTN registry (ISRCTN16849867) on 6 March 2017.

## Background

Third molar surgery is one of the most common operations in oral and maxillofacial surgery. Pain, swelling, and trismus are the most common symptoms that affect patients’ quality of life. Alveolitis, infection, and hemorrhage are common complications [1, 2]. Many attempts have been made to reduce the risk of complications and improve patients’ quality of life, such as platelet-rich plasma (PRP) or platelet-rich fibrin (PRF) administration [3, 4], lasers [5], cryotherapy [6], drug therapies [7], and osteotomy or flap designs [8, 9]. However, the exact solution for pain and edema has not yet been found.

PRF clots, developed by Chouckroun et al. [10], are comprised of platelets, leucocytes, cytokines, and circulating stem cells that are enmeshed by a fibrin matrix [10]. These components make PRF a healing biomaterial that permits optimal healing [11]. PRF belongs to a next generation of platelet concentrate geared to simplified preparation without biochemical blood handling [12]. Extraction sockets would heal more quickly and pain would be reduced if autogenous platelet concentrate was applied to the area. [10] Many studies showed that PRF accelerated wound healing in periodontal defects, cyst cavities and sinus augmentations [10, 13, 14].

The aim of the study was to evaluate the effects of PRF on postoperative pain and edema after third molar surgery.

## Methods

This study was conducted at the Ankara University Faculty of Dentistry, Department of Oral and Maxillofacial Surgery from September 2012–May 2013. Thirty patients (21 male, 9 female) aged 17–27 years were selected for removal of bilaterally impacted mandibular third molars. The local ethical committee of the Ankara University Faculty of Dentistry approved the study protocol (Date-number: 28.11.2011 - 25/1). All of the patients were informed of the nature of the surgical and experimental procedures, and their informed consent was obtained before surgery.

### Inclusion criteria

- Patients who fit the study requirements including follow up coming sessions and informed consent signing
- Healthy patients without significant medical diseases or a history of bleeding problems
- Patients’ impacted third molars had to be symmetrical and feature the same level of surgical difficulty that required the same surgical technique to be performed
- The third molars had to be in the Class I, Level B position (according to Pell &Gregory) and in the vertical positions according to Winter.

### Exclusion criteria

- Pregnant and lactating women
- Patients with signs of pericoronitis
- Patients with chronic use of medications such as antihistamines, non steroidal anti inflammatory drugs (NSAID, steroids and antidepressants which would complicate the evaluation of their postoperative response.

Bilateral removal of the third molar was performed in a single appointment. For the study side, the sockets were filled with PRF, whereas for the control side, the sockets were left empty. The study sides and control sides were selected randomly.

### Preparation of PRF

Before surgery, a 3 x 10 ml blood tube (BD VACUTAINER) with clot activator was used to obtain blood from either the cephalic or basilica vein of each patient with a vacutainer needle. The blood samples were immediately centrifuged at 3,000 rpm for 10 min (NUVE NF 200, Turkey). After centrifugation, the PRF was gently seperated from red corpuscles.

### Surgical procedure

An experienced oral surgeon performed the surgical extraction with a standardized technique. Patients did not use any preoperative anti-inflammatory or antimicrobial drugs. Inferior dental and buccal nerve anesthesia was applied using a solution of 4% articaine hydrochloride and 1:100,000 epinephrine. A triangular full thickness flap with releasing incision on the disto-buccal aspect of the second molar was used. Bone removal was done with round bur. After exposing the tooth, if necessary, tooth sectioning was performed; then, the tooth was extracted with an elevator. After extraction, granulation tissue, follicular remnants, and bony spicules were removed from the socket, which was then irrigated with an isotonic saline solution. On the study side, the socket was filled with three pieces of PRF membrane, and the flap was primarily closed with 3–0 silk sutures. Pressure packs were applied. The sutures were removed on postoperative day seven. Amoxicillin (1000 mg twice per day for five days), %0.2 chlorhexidine mouthwash (twice per day for seven days) and if necessary, acetaminophen (500mg up to four times per day) were prescribed postoperatively.

### Evaluation procedure

Facial swelling was determined by measuring distances from gonion- comissura labiorum, tragus – comissura labiorum and tragus – lateral canthus. Measurements were performed with a flexible ruler preoperatively and postoperatively day 2 and 7. For standardization all measurements were performed by the same surgeon (UG).

Patients’ pain was evaluated with a line-type visual analogue scale (VAS) and a verbal scale (VRS). All of the patients completed the VAS to assess their pain, with endpoint-marked scores of 0 (no pain) to 100 (worst pain) and VRS with scores of 0 (no pain) to 5 (intolerable pain).

### Statistical analysis

SPSS version 20.0 was used for the statistical analysis. The pain values had an abnormal distribution; on the contrary, the edema values had a normal distribution. The preoperative and postoperative pain values between the sides were compared with the Mann–Whitney U test (*p* < 0.05). Preoperative and postoperative edema values between the groups were compared with independent sample t-tests (*p* < 0.05).

In both groups, the preoperative and postoperative pain values between the follow-up periods were compared using the Wilcoxon sign test (*p* < 0.05). Preoperative and postoperative edema values between the follow-up periods were compared with paired-sample t-tests (*p* < 0.05).

## Results

The study included a total of 30 patients aged 17–27 years (mean age = 20.03). Nine patients were male (30%), and 21 patients were female (70%). Uneventful recovery occurred in 27 patients; however, infection was observed in three patients who were treated without PRF.

Pain values measured with the VAS decreased in both groups; however, there were no statistically significant differences between the groups (Table 1). Pain values measured with the VRS decreased in both groups; however, there were no statistically significant differences between the groups (Table 2).

**Table 1:** Pain evaluation between groups (VAS)

		Group					Mann Whitney U Test		
		n	Mean	Min	Max	SD	Mean Rank	U	P
6 hour	PRF+	30	42.7	3.0	98.0	27.5	31.4	424.5	0.706
	PRF-	30	40.0	0.0	96.0	26.3	29.7		
	Total	60	41.3	0.0	98.0	26.7			
12 hour	PRF+	30	36.1	0.0	99.0	28.5	32.5	390	0.374
	PRF-	30	30.0	0.0	100.0	28.9	28.5		
	Total	60	33.0	0.0	100.0	28.6			
1st day	PRF+	30	25.0	0.0	99.0	26.3	32.4	393.5	0.398
	PRF-	30	20.9	0.0	83.0	26.1	28.6		
	Total	60	23.0	0.0	99.0	26.0			
2nd day	PRF+	30	15.8	0.0	100.0	20.9	31.5	420.5	0.655
	PRF-	30	13.8	0.0	69.0	18.4	29.5		
	Total	60	14.8	0.0	100.0	19.6			
3rd day	PRF+	30	7.9	0.0	51.0	12.1	30.2	439.5	0.864
	PRF-	30	8.0	0.0	42.0	12.3	30.9		
	Total	60	8.0	0.0	51.0	12.1			
7th day	PRF+	30	1.0	0.0	12.0	3.0	31.0	434.5	0.681
	PRF-	30	0.8	0.0	11.0	2.7	30.0		
	Total	60	0.9	0.0	12.0	2.8

**Table 2:** Pain evaluation between groups (VRS)

		Group					Mann Whitney U Test		
		n	Mean	Min	Max	SD	MeanRank	U	p
6 hour	PRF+	30	2.30	0.00	4.00	1.12	31.32	425.5	0.709
	PRF-	30	2.20	0.00	5.00	1.21	29.68		
	Total	60	2.25	0.00	5.00	1.16			
12 hour	PRF+	30	2.07	0.00	5.00	1.26	32.20	399	0.436
	PRF-	30	1.83	0.00	5.00	1.29	28.80		
	Total	60	1.95	0.00	5.00	1.27			
1st day	PRF+	30	1.33	0.00	5.00	1.24	31.05	433.5	0.800
	PRF-	30	1.27	0.00	4.00	1.26	29.95		
	Total	60	1.30	0.00	5.00	1.24			
2nd day	PRF+	30	1.10	0.00	5.00	1.12	30.97	436	0.827
	PRF-	30	1.07	0.00	5.00	1.14	30.03		
	Total	60	1.08	0.00	5.00	1.12			
3rd day	PRF+	30	0.53	0.00	2.00	0.68	30.95	436.5	0.820
	PRF-	30	0.50	0.00	2.00	0.68	30.05		
	Total	60	0.52	0.00	2.00	0.68			
7th day	PRF+	30	0.10	0.00	1.00	0.31	31.00	435	0.643
	PRF-	30	0.07	0.00	1.00	0.25	30.00		
	Total	60	0.08	0.00	1.00	0.28			

In both groups, postoperative edema increased significantly in the first two days post-surgery. Postoperative edema values at postoperative day one were significantly lower than postoperative day two. However, there were no significant differences between the groups (Table 3).

**Table 3:** Edema evaluation between groups

		Group					Independent samples test	
		n	Mean	Min	Max	SD	t	p
Gonion- commissura_preop	PRF+	30	8.7	7.1	10.8	0.8	1.032	0.306
	PRF-	30	8.5	6.5	9.7	0.8		
	Total	60	8.6	6.5	10.8	0.8		
Gonion- commissura_2nd day	PRF+	30	9.5	7.8	11.7	1.0	-0.089	0.930
	PRF-	30	9.5	7.8	10.6	0.8		
	Total	60	9.5	7.8	11.7	0.9		
Gonion- commissura_7.th day	PRF+	30	8.8	7.2	11.0	0.8	0.834	0.408
	PRF-	30	8.7	6.6	9.9	0.8		
	Total	60	8.8	6.6	11.0	0.8		
Tragus - commissura_preop	PRF+	30	10.8	9.0	12.4	0.8	0.278	0.782
	PRF-	30	10.8	9.5	12.3	0.7		
	Total	60	10.8	9.0	12.4	0.8		
Tragus - commissura_2nd day	PRF+	30	11.3	9.7	13.5	0.9	-0.328	0.744
	PRF-	30	11.3	10.0	12.9	0.7		
	Total	60	11.3	9.7	13.5	0.8		
Tragus - commissura_7th day	PRF+	30	10.9	9.2	12.4	0.8	0.197	0.845
	PRF-	30	10.8	9.7	12.3	0.7		
	Total	60	10.9	9.2	12.4	0.8		
Gonion-lateral canthus_preop	PRF+	30	9.8	8.3	11.8	0.8	0.730	0.468
	PRF-	30	9.7	8.2	11.5	0.8		
	Total	60	9.7	8.2	11.8	0.8		
Gonion- lateral canthus_2nd day	PRF+	30	10.1	8.3	12.0	0.8	0.283	0.778
	PRF-	30	10.0	8.4	11.8	0.7		
	Total	60	10.0	8.3	12.0	0.8		
Gonion- lateral canthus_7th day	PRF+	30	9.7	4.0	11.8	1.3	-0.131	0.896
	PRF-	30	9.7	8.2	11.5	0.8		
	Total	60	9.7	4.0	11.8	1.1		

## Discussion

There is a very limited amount of literature on the effect of PRF on pain and swelling in third molar surgery. The aim of the present study was to investigate the effect of PRF application on postoperative pain and edema after the surgical removal of mandibular third molars. The null hypothesis was that postoperative pain and edema with and without PRF after surgery would be equal. The authors measured and compared postoperative pain and edema after the surgical removal of impacted mandibular third molars in PRF and non-PRF sockets.

PRF is the second generation of platelet concentrates (PRP is the first generation). PRF contains various autologous cytokines and immune cells; it is a fibrin membrane that covers the wound appropriately and can be sutured [15].

In the oral and maxillofacial region, PRF has been widely used in sinus augmentation as the sole grafting material or in combination with an allograft or a xenograft. [16] PRF clots are also used for the flapless treatment of acute sinus perforations [17]. Extraction socket preservation, intrabony defects, and periodontal problems are the other indications of intraoral PRF usage [11].

In a study of 31 patients Kumar et al. [18] reported that PRF usage decreased pain and swelling values significantly on the first control day post surgery. They recorded these values using a Likert type VAS as required by Pasqualini et al. [19].

In an another study conducted on 20 bilateral impacted mandibular third molar surgeries, Singh et al. [20] reported that PRF usage after third molar surgery decreased pain in the first, third, and seventh days post-surgery (measured with a Likert-type VAS); however, this finding was not statistically significant.

In a multicenter study with a large sample (56 patients, 102 teeth), Özgül et al. [21] reported that using PRF after third molar extraction significantly decreased horizontal swelling (involving tragus and commissura measurement) on the first and third day post-surgery. They stated that no significant differences were observed in the seventh day post-surgery. They also found no significant differences in vertical swelling, which involved lateral canthus and gonion measurement, or pain at all intervals. Overall, the authors reported that bilateral operation in the same session could affect the pain measurement conducted with a line-type VAS.

In a study containing 59 patients, Bilginaylar et al. [22] reported that PRF usage decreased pain values significantly on the first, third, and seventh days post-surgery. They evaluated pain with a line-type VAS. However, unlike Kumar et al. [18], there were no significant differences in swelling values on the first day post-surgery. They also specified that no significant differences were found on the third and seventh days post-surgery. They stated that tape measurement could be the reason for the different swelling scores.

Uyanık et al. [4] extracted impacted third molars bilaterally in 20 patients and reported that PRF usage in impacted third molar surgery reduced pain significantly on the first, second, third, and seventh days post-surgery (pain was evaluated with a Likert-type VAS). However, no significant differences were found regarding swelling, which was evaluated via tape measurement [4].

In another study comprised of 30 patients, Asutay et al. [23] reported that no significant differences were observed between the PRF and control groups at all intervals due to improvement of pain and swelling values. This study used 3dMD to evaluate swelling, while a Likert-type VAS was used to evaluate pain. They reported that all of the operations were done in a series of two appointments [23].

Gürler et al. [24] reported that Leukocyte PRF(L- PRF) application to the impacted mandibular third molar extraction sockets in 40 patients was not found statistically significant in terms of postoperative pain and edema. They stated that pain evaluated with a Likert type VAS scale whereas edema evaluated with flexible ruler [24].

Our study involved 30 patients who underwent bilateral third molar surgery in the same session. Bilateral operations in the same sessions may have influenced pain results [21]. To make an objective evaluation, pain values were evaluated with a line-type VAS and VRS; however, no significant differences were observed between the groups according to both scales at all intervals. Results are in accordance with Singh et al. [18], Özgül et al. [21], Asutay et al. [23] and Gürler et al. [24]. A flexible tape scale was used to cheaply and effectively measure facial edema. However we found that PRF had no significant effect on edema at all intervals. These findings are similar with Bilginaylar and Uyanık [22], Uyanık et al. [4], Asutay et al. [23] and Gürler et al. [24].

## Conclusions

PRF had no significant effect on swelling and pain after lower third molar surgery, compared to the healing without it. To obtain more meaningful results, future research should use a larger sample with different evaluation methods for all variables (i.e., pain and swelling).

## Abbreviations

PRF: Platelet rich fibrin
PRP: Platelet rich plasma
VAS: Visual analogue scale
VRS: Verbal rate scale
NSAİD: Non steroidal anti inflammatory drugs
